# Improving Delirium Screening and Management in a Hospice Inpatient Unit: Audit and Quality Improvement Cycles Using the 4AT (Alertness, Abbreviated Mental Test-4 (AMT4), Attention, Acute Change) Tool

**DOI:** 10.7759/cureus.97761

**Published:** 2025-11-25

**Authors:** Isabel Brewster, Lucy Anson, Rachel Clingan, Aruni Wijeratne

**Affiliations:** 1 Hospital-based Medicine, Epsom and St Helier University Hospitals NHS Trust, London, GBR; 2 Hospital-based Medicine, Imperial College Healthcare NHS Trust, London, GBR; 3 Palliative Medicine, Princess Alice Hospice, London, GBR

**Keywords:** 4at tool, delirium, documentation, electronic medical records, hospice, palliative care

## Abstract

Introduction: Delirium is highly prevalent in palliative care settings, contributing to patient distress, poorer outcomes, and increased mortality. Although validated screening tools exist, many hospices in the UK rely on clinical judgement alone. In this study, we evaluated the use of the 4AT (Alertness, Abbreviated Mental Test-4 (AMT4), Attention, Acute Change) delirium screening tool on admission to a hospice inpatient unit (IPU) and implemented quality improvement (QI) interventions to enhance subsequent documentation and management.

Methods: A retrospective audit of 25 admissions to the IPU at Princess Alice Hospice in London, UK, in August 2023, assessed 4AT completion rates, resultant scores, documentation of contributing factors, and clinical actions taken. The findings informed a QI project employing two Plan-Do-Study-Act (PDSA) cycles. Interventions included modifying the electronic medical record (EMR) template, embedding a delirium clinical guide, and educating clinicians. Anonymous questionnaires gathered feedback on usability and clinician confidence before and after the interventions.

Results: In the audit, 88% of patients had 4AT assessments completed, with 36% scoring ≥4 (suggesting possible delirium). No explicit causes or structured action plans were recorded, despite the implication of contributing factors. Following QI interventions, staff reported that the modified EMR template had improved their documentation and was user-friendly. Clinician confidence in managing patients with high 4AT scores increased: 75% of respondents felt "very confident" post-intervention compared with 14% at baseline.

Discussion: The 4AT screening tool can be effectively integrated into hospice admission workflows. Targeted EMR modifications and clinician education improved documentation and confidence, supporting the better identification and management of delirium. Limitations include small sample size, single-site design, and reliance on qualitative staff feedback.

Conclusions: Embedding a structured screening tool within EMR, supported by clinical guidance and education, enhances the assessment and management of delirium in hospices.

## Introduction

Delirium is a complex neuropsychiatric syndrome characterised by fluctuating cognition, inattention, and altered awareness. It is highly prevalent in palliative care patients due to multiple factors, including the effects of medications, advanced age, organ failure, and underlying medical conditions. Delirium increases morbidity, mortality, and distress for patients and families. It is important to screen for delirium because early detection leads to prompt treatment and potential reversal, improving patient outcomes and reducing anxiety for patients and relatives. In a systematic review in 2019, researchers reported that one-third of adults had a delirium on admission to a palliative care setting, which increased to two-thirds who developed delirium during the admission [1].

Despite this finding, delirium screening in UK hospices is inconsistent. Only 13% of palliative care teams routinely use a recognised screening tool on initial inpatient presentation [2]. Instead, assessment is often down to clinical judgement, risking under-detection. The Scottish Intercollegiate Guidelines Network [3] recommends validated screening tools, including the 4AT (Alertness, Abbreviated Mental Test-4 (AMT4), Attention, Acute Change), a rapid bedside test to identify delirium and cognitive impairment.

At the hospice, the 4AT was incorporated into the admission clerking bundle on the electronic medical record (EMR) but was not a mandatory template. Previous quality improvement (QI) work at the hospice highlighted a high prevalence of delirium and recommended the formal integration of 4AT. This project aimed to raise awareness of delirium prevalence, evaluate current screening practice through 4AT completion rate, identify gaps in documentation and management actions, and implement improvements using QI methodology.

A version of this work was previously presented as a poster and published as a conference abstract in BMJ Support Palliat Care (2024, 14:A69.3).

## Materials and methods

Design

We conducted a two-stage project: first, an audit of admissions to evaluate delirium screening and management, and second, a QI project with two Plan-Do-Study-Act (PDSA) cycles to improve documentation and clinical response. The study was conducted at Princess Alice Hospice in London, UK. Consecutive patients admitted up to August 31, 2023, were included retrospectively (n = 25). All had cancer as their primary diagnosis.

Audit methods

Retrospective evaluation of documentation on the EMR assessed the rate of 4AT completion on admission and the interpretation of 4AT scores. For those with a significant score indicating possible delirium, further evaluation of the EMR followed for the identification of potential causative factors and actions taken as management. Admission outcome for patients, either discharge or death, was also recorded. The standard set for the audit stated that 100% of patients should have a 4AT completed on admission or a reason documented as to why it was not completed.

QI methods

The local IDEAS (Involve, Design, Execute, Analyse, Share) framework guided the QI process. Two PDSA cycles were undertaken.

PDSA 1 included modifications to the EMR template with free-text boxes added, the delirium clinical guide embedded, and mandatory fields included to document likely aetiology and action plans for significant scores of 4 and above. Teaching was provided for end users (hospice doctors) during a one-hour tutorial session, and a questionnaire was administered to assess baseline knowledge.

PDSA 2 included the simplification of the EMR template based on feedback and a repeat questionnaire for clinician feedback.

Anonymous questionnaires assessed usability and clinician confidence. Data was analysed descriptively.

## Results

Audit findings

With regard to the patient population, of the 25 patients, 22 (88%) were admitted for terminal care, and 21 (84%) died during admission. The rate of 4AT completion was 22/25 (88%). Two were not completed because the patients were unresponsive, and one tool was incomplete. In terms of 4AT scores, 8/22 (36%) had scores ≥4 indicating possible delirium ± cognitive impairment. The breakdown of significant scores included 12 (n = 3), 8 (n = 3), 7 (n = 1), and 4 (n = 1). Two patients scored 2, and 12 patients scored 0. With regard to documentation, no cases had explicit causes of delirium recorded. Further retrospective evaluation of EMR following a significant 4AT score in eight patients showed that multiple contributing factors including pain, nicotine withdrawal, hypercalcaemia, and constipation were identified and implied but not labelled explicitly as the potential causes of delirium. Actions were also inconsistently linked throughout (Table 1).

**Table 1:** Audit results of 25 admissions 4AT: Alertness, Abbreviated Mental Test-4 (AMT4), Attention, Acute Change

Variable	n (%)
4AT completed	22 (88%)
4AT ≥4	8 (36%)
Explicit cause documented	0 (0%)
Action taken linked to score	1 (12.5%)
Died during admission	21 (84%)

Outcomes

All patients with scores ≥4 died during admission (mean 10.6 days).

PDSA cycle 1 findings

**Figure 1:**
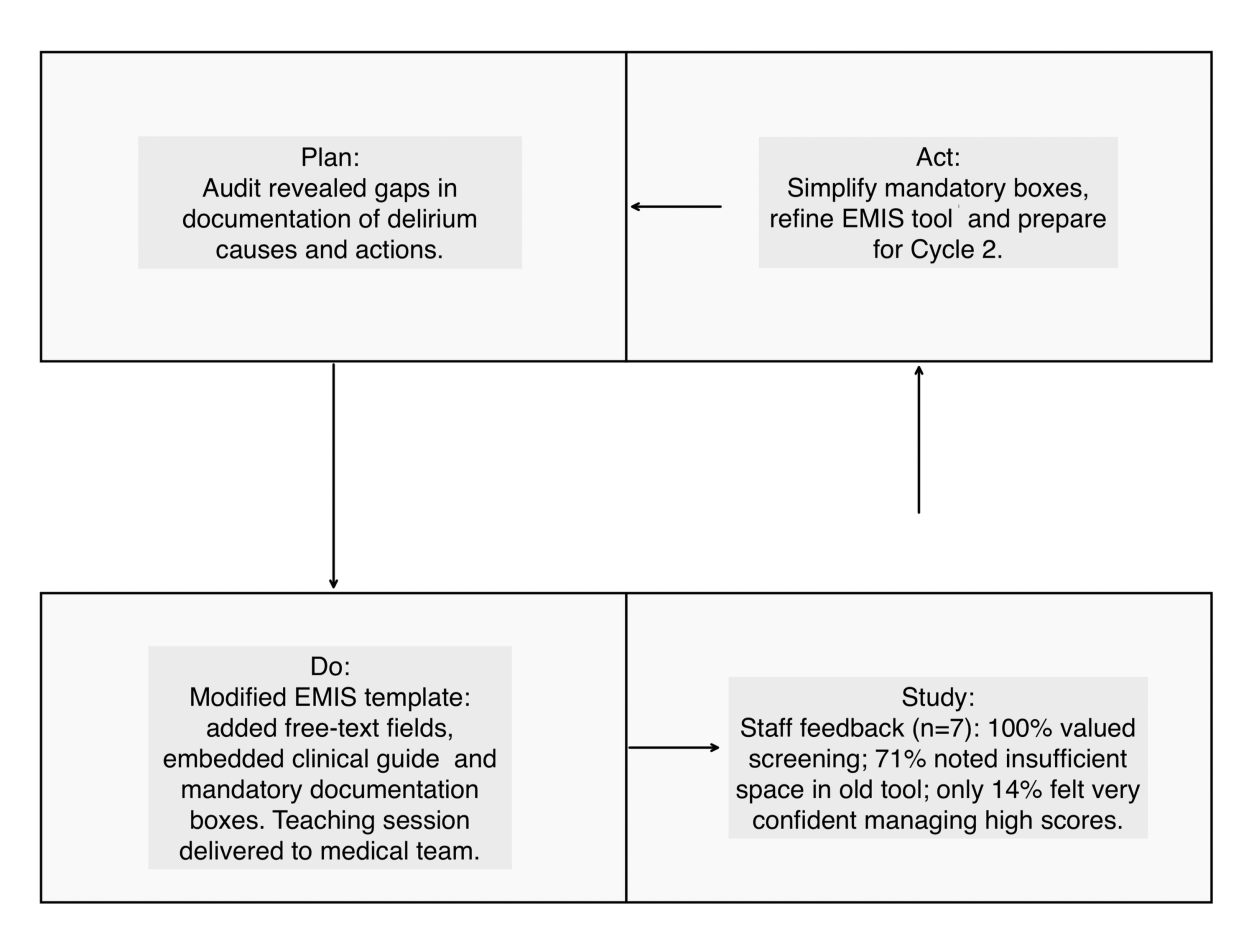
PDSA cycle 1: template changes and baseline staff feedback EMIS: Education Management Information System; PDSA: Plan-Do-Study-Act

Interventions introduced free-text boxes onto the EMR template, embedded a clinical delirium guide, and included mandatory boxes for "assessment of causes" and "need for review" only for patients with a significant 4AT score of 4 and above. We also delivered a one-hour teaching session to the medical team which included key findings from the audit, education surrounding the importance of delirium screening, and discussion about EMR template changes (see Figure 1).

Feedback from clinicians (n = 7) recorded that 100% said 4AT was important for patients. Around 85% said the original tool was user-friendly; however, 71% said they were unable to document fully with the old template. In terms of clinician confidence in actioning a significant 4AT score, 14% reported being "very confident", 57% reported moderate confidence, and 29% reported low confidence.

Concerns were raised regarding the necessity of multiple mandatory boxes.

PDSA cycle 2 findings

**Figure 2:**
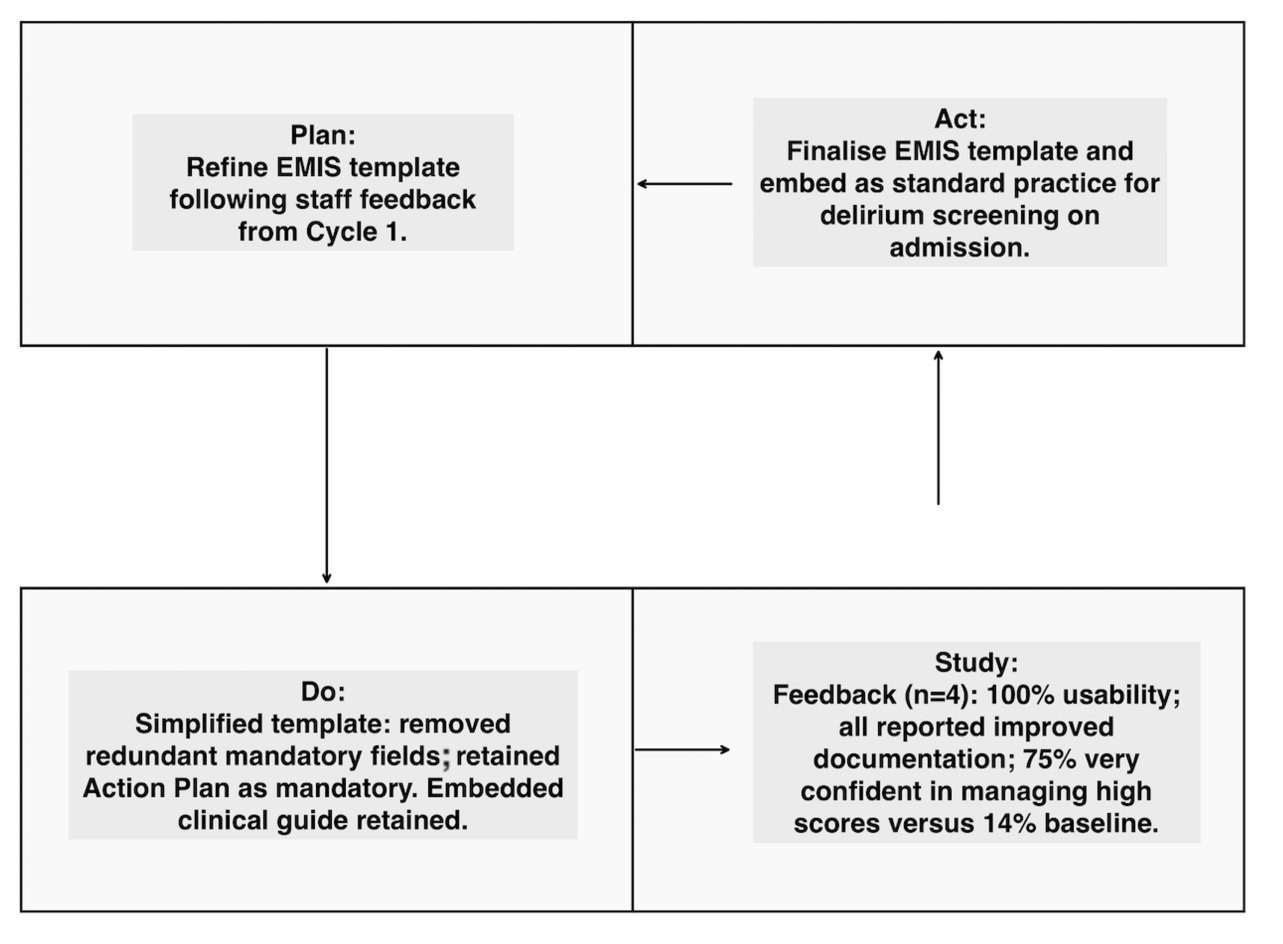
PDSA cycle 2: template refinement and improved clinician confidence EMIS: Education Management Information System; PDSA: Plan-Do-Study-Act

Interventions simplified the EMR template which removed redundant mandatory boxes for "assessment of causes" and "need for review" but retained the "action plan" box as a mandatory field (see Figure 2).

Feedback from clinicians (n = 4) recorded that 100% said the new tool was user-friendly and 100% said the documentation had improved. About 75% felt "very confident" managing significant scores (vs 14% at baseline). All found the embedded delirium clinical guide (see Table 2) helpful.

**Table 2:** Delirium clinical guide MI: myocardial infarction; RR: respiratory rate; Abdo: abdomen; CNS: central nervous system; PR: per rectal examination; MSU: midstream urine; CSU: catheter specimen of urine; FBC: full blood count; U&Es: urea and electrolytes; LFTs: liver function tests; Ca: calcium; Alb: albumin; CRP: C-reactive protein; TFT: thyroid function test; ECG: electrocardiogram; CXR: chest X-ray; CT: computed tomography; PD: Parkinson's disease; DOLS: Deprivation of Liberty Safeguards

Causes of delirium	
D	Drugs (sedatives, opiates, antidepressants, anticholinergics, polypharmacy)
E	Electrolyte imbalance/endocrine/environment change (dehydration, hypo-/hyperglycaemia, hypercalcaemia, renal/liver failure, vitamin deficiency (B12, thiamine, folate), thyroid, temperature, room change)
L	Lack of drugs (alcohol, nicotine, other, uncontrolled pain)
I	Infection/intercurrent illness
R	Reduced sensory input (vision/hearing loss)
I	Intracranial (stroke, subdural, brain metastasis, post-ictal)
U	Urinary retention/faecal impaction
M	Myocardial/pulmonary (MI, angina, pulmonary embolism, hypoxia, hypotension, anaemia)
Check observations (temp, pulse, RR, blood glucose)	
Full examination (chest, Abdo, skin, CNS, +/- PR, bladder scan)	
Signs of withdrawal from drugs, alcohol, nicotine	
Evidence of pain	
Urine dip +/- MSU/CSU	
Sputum culture	
Review medications	
Bloods: FBC, U&Es, LFTs, Ca, Alb, glucose, CRP; consider TFT and B12/folate	
Consider hospital Ix: ECG, CXR, head CT	
Regular observations	
Treat any underlying cause	
Stop or reduce contributing drugs	
Good sensory environments (glasses/hearing aids, well-lit room during the day, reduce noisy environments, low-level light at night)	
Educate and support the family	
Regular orientation (clocks/calendars, visitors, familiar objects/photos)	
Promote mobility where safe to do so	
Staff continuity when possible	
Reduce the need for wandering	
Restore sleep pattern: use non-pharmacological means where possible	
Pharmacological management only when necessary; haloperidol (not if PD or Lewy body dementia) +/- lorazepam lowest dose for the shortest time	
Is DOLS needed?	

## Discussion

This project confirms that a validated tool such as the 4AT (a rapid delirium screening instrument) can be feasible for routine use in a hospice inpatient unit, with a high completion rate (88%) and a significant proportion of patients scoring ≥4 (36%). The diagnostic accuracy of the 4AT has been well established; in a meta-analysis of 17 studies (3,702 observations), it achieved a pooled sensitivity of approximately 0.88 and specificity of 0.88 [4]. The ease of use without extensive training makes it particularly suitable for clinical settings where rapid assessment is needed.

However, our audit revealed that, despite frequent screening, explicit documentation of possible causes and linked actions following a significant 4AT score was absent. This suggests that screening alone, without structured follow-up, may fail to translate into meaningful clinical change.

QI and EMR integration

The subsequent QI project, using two sequential PDSA cycles, addressed this gap by introducing the 4AT as mandatory within the EMR admission workflow, adding embedded guidance and adapting the template based on user feedback. Implementation of digital workflow interventions has previously been shown to improve clinician adherence and documentation quality; for example, a QI programme to improve EMR documentation found measurable improvements in adoption and workflow compliance [5]. Additionally, electronic health record-based behavioural change interventions are increasingly recognised as potent levers for altering clinician behaviour when informed by behavioural science frameworks [6]. The structured process of auditing, designing change, executing templates, and gathering feedback mirrors the "audit-feedback" loop that has been repeatedly associated with modest but meaningful improvements in professional practice [7].

By involving end users (hospice doctors) at every stage, our approach turned a tool (the 4AT) into an integrated system. Doctors reported that the refined template was "user-friendly" and enabled better linkage between the screening result and clinical action. Therefore, clinician confidence in managing identified delirium rose from 14% to 75% feeling "very confident". This aligns with evidence that clinician behaviour change is more likely when systems support action, reduce cognitive load, and embed prompts at the point of care. The two PDSA cycles reflect the adaptive nature of QI: PDSA 1 identified design issues and staff concerns, whilst PDSA 2 implemented refinements and achieved higher usability and confidence.

Impact on clinician behaviour

The key success of this project lies not only in screening uptake but also in shifting clinical practice: documentation improved, screening became embedded, and action planning was more explicit. The workflow's human-factors design (reducing complexity, improving visibility of prompts, and linking action to screening) is consistent with literature on behaviour change in healthcare professionals. Systematic reviews show that multifaceted interventions (e.g., audit with feedback, digital prompts, and education) are more effective than single-mode interventions [8]. In our case, the digital template acted as both a prompt and a documentation vehicle, which arguably fosters sustained behavioural change rather than one-off compliance.

The combination of validated screening (4AT), embedded support (EMR template), and clinician engagement provides a model for bridging the gap between evidence and practice, a gap widely recognised in healthcare [9]. By converting screening into actionable workflows and tying them into admission processes, the intervention facilitated the translation of evidence into routine practice.

Implications for practice

The findings indicate that combining robust screening tools with system redesign and user involvement can effect meaningful practice change in palliative care settings. Adoption of the 4AT alone may not suffice: ensuring workflow integration, linking screening to action, and engaging staff in iterative improvement are keys. For hospices and palliative care services wishing to improve delirium detection and management, this model suggests a feasible pathway. By leveraging digital tools and human-factors design, teams can enhance adherence, streamline documentation, and improve clinician confidence. 

Implications for patient and family outcomes

This project has meaningful implications for patient and family outcomes within the hospice setting. Early recognition of delirium enables more timely identification of potentially reversible causes, which can reduce distress, improve patient comfort, and support clearer communication with relatives. By increasing clinician confidence and standardising documentation, the project strengthens the likelihood that delirium is actively managed rather than accepted as part of terminal decline.

Limitations

Nevertheless, the project has limitations. It was a single centre, with a relatively small sample size, limiting generalisability. Additionally, although the principles of the intervention are reproducible across similar settings, full reproducibility would require clearer operational details. The outcomes measured were process-based (screening completion, documentation, and clinician confidence) rather than patient-centred (delirium incidence reduction, symptom control, and family satisfaction). There are barriers present during admission to hospice which may limit assessment including patient distress or significant symptom burden. In some cases, 4AT is not appropriate to complete altogether. Screening accuracy of the 4AT is established in other settings; however, its performance and patient-outcome impact in hospice populations remain less well studied [4]. Further, the QI intervention may be affected by context (EMR system or local culture) and may require adaptation elsewhere.

## Conclusions

Delirium is highly prevalent in hospice inpatients but remains under-recognised and under-documented. This project demonstrates the successful integration of a validated delirium screening tool, the 4AT, into hospice clinical practice through a structured and collaborative QI approach. The initial audit identified key shortcomings in documentation and the translation of screening outcomes into action, underscoring the gap between the recognition and management of delirium in palliative settings. Across two iterative PDSA cycles, clinician feedback guided adjustments to the EMR template creating a practical structure for consistent delirium assessment and management. This collaborative approach ensured that interventions were acceptable to users and led to measurable improvements in documentation quality and clinician confidence whilst managing delirium. This enhances patient and family outcomes by enabling earlier recognition and more consistent management of delirium, thereby reducing distress and improving the quality of care. 

The success of this process highlights the power of user involvement and continuous feedback in translating evidence-based tools into effective, real-world practice to improve clinical care. This project reinforces the broader lesson that sustained QI in healthcare depends on clinician ownership, practical system design, and continuous adaptation. By aligning digital tools with everyday clinical practice, we were able to move from reactive to proactive delirium care. The lessons learned here extend beyond delirium screening: they illustrate how thoughtful, user-centred QI can strengthen care delivery, enhance confidence among doctors, and ultimately improve patient outcomes in hospice settings.
